# Benzimidazole derivatives as a new scaffold of anticancer agents: Synthesis, optical properties, crystal structure and DFT calculations

**DOI:** 10.1016/j.heliyon.2024.e32905

**Published:** 2024-06-13

**Authors:** Mohamed Oussama Zouaghi, Donia Bensalah, Sabri Hassen, Youssef Arfaoui, Lamjed Mansour, Namık Özdemir, Hakan Bülbül, Nevin Gurbuz, Ismail Özdemir, Naceur Hamdi

**Affiliations:** a Laboratory of Characterizations, Applications & Modeling of Materials (LR18ES08), Department of Chemistry, Faculty of Sciences of Tunis, University of Tunis El Manar, 2092, Tunisia; b Research Laboratory of Environmental Sciences and Technologies (LR16ES09), Higher Institute of Environmental Sciences and Technology, University of Carthage, Hammam-Lif, Tunisia; c Zoology Department, College of Science, King Saud University, Saudi Arabia, P.O. Box 2455, Riyadh, 11451, Saudi Arabia; d Department of Physics, Faculty of Science, Ondokuz Mayıs University, 55139, Samsun, Turkey; e İnönü University, Faculty of Science and Art, Department of Chemistry, Malatya, 44280, Turkey; f İnönü University, Catalysis Research and Application Center, Malatya, 44280, Turkey

**Keywords:** Benzimidazole derivatives, Density functional theory (DFT), UV absorption, A8/nticancer evaluation, Cytotoxicity assessment

## Abstract

The absolute necessity to fight some class of tumour is perceived as serious health concerns, and the discovery and development of effective anticancer agents are urgently needed. So, the novel benzimidazole derivatives (**2a-b**) were designed, synthesized, with their structures rigorously characterized using single X-ray crystallography, FT-IR, UV, and NMR spectroscopy, alongside elemental analysis. The geometric structures were optimized using density functional theory (DFT) calculations performed at the ωB97X-D/cc-pVDZ level, yielding good agreement with experimental XRD data. The studied salt complexes exhibited the ability to absorb UV light at 275 nm. Furthermore, anticancer activity of the compounds was screened against (*MDA-MB-231*, *MCF-7*, *HT-29* and healthy cell line (*HF*)) and revealed the remarkable efficacy of select newly synthesized Benzimidazole derivatives (**2a-b**). Compound **2a** showed relative significant higher cytotoxicity (165.02) in *MDA-MB-231* cancer cell line. This underscores their promising potential in therapeutic applications, affirming their role as valuable contenders in the pursuit of novel anticancer agents.

## Highlights

• Novel benzimidazole derivatives (**2a-b**) were designed, synthesized.
• Their structures were confirmed using single X-ray crystallography, FT-IR, UV, and NMR spectroscopy, alongside elemental analysis.
• Furthermore, anticancer activity of the compounds was screened against (*MDA-MB-231*, *MCF-7*, *HT-29* and healthy cell line (*HF*)) and revealed significant higher cytotoxicity.

## 1 Introduction

**Fig. 1:**
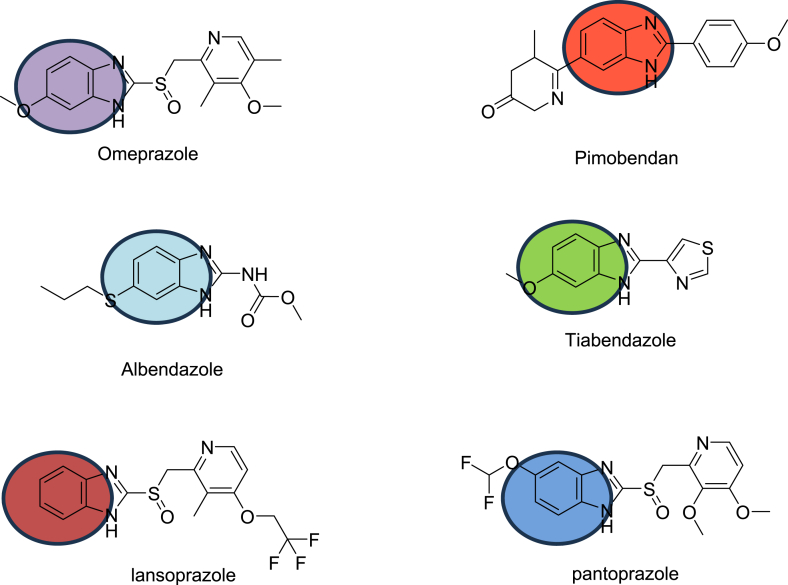
Biologically active drug molecules containing benzimidazoles like omeprazole (used for gastroesophageal diseases), pimobendan (vasodilator), albendazole (parasitic worm infestations), tibendazole, (antifungal agent), lansoprazole (Anti acid) and pantoprazole (proton pump inhibitor) [[1], [2], [3], [4], [5], [6],^9^,^10^].

Benzimidazoles are well known nitrogen-containing bioactive compounds. They showed a variety of activities including anti-diabetic [1], antimicrobial [2], antifungal [3], antiviral [4], anti-cancer activity [[5], [6], [7]] (including anti breast cancer [8]) and anthelminthic [9]. Recent studies in medicinal chemistry have demonstrated them as potential drug motifs for the pharmaceutical industry [10]. They have also well-known applications in chemo-sensing [11], fluorescence [12], crystal engineering [13], corrosion science [14,15] and asymmetric catalysis [16]. The bioactivity of drug molecule depends predominantly on their interaction with drug targets, such as proteins, enzymes, nucleic acids and the biological membranes [17,18]. The efficacy and of the imidazole can be impute to its H-bond donor and acceptor ability as well as its binding affinity with metals [19] (see Fig. 1).

On the other hand, in order to achieve highly active new spices, the research on the structural

Modification and improvement of the existing varieties is still challenging in the area of materials. To get more detailed information about this kind of compounds and therefore to broaden their application, we are here providing experimental and theoretical research on a predesigned benzimidazobenzimidazole compound, focusing on its preparation, characterization and optical properties. As we know, many luminescence materials exhibit high fluorescence quantum yield in solution, but become either weakly or even not emissive at all in the solid state because of self-quenching caused by π-π stacking interactions. Many studies show that greater steric congestion can effectively reduce π-π stacking interactions between the dye molecules to avoid concentration quenching [[9], [10], [11]]. On the other hand, introduction of a fluorine atom into organic molecules leads to compounds with strongly different properties due to high electronegativity of the fluorine atom and its small size. For example, many fluorinated organic compounds were reported having special electronic and optoelectronic properties [[12], [13], [14], [15]]. Infrared resonance (IR) and nuclear magnetic resonance (NMR) techniques are the most powerful methods for the structural identifications of organic compounds and interpretations of their chemical behaviors [[16], [17], [18]].

Thus, the aim of this study is to synthesize novel unsymmetrical benzimidazole derivatives **(2a-b)** with various combinations on their nitrogen atoms, and characterized by IR, ^1^H NMR and ^13^C NMR. The optical properties including UV–vis absorption and fluorescence emission spectra were investigated experimentally. Additionally, a DFT and TD-DFT study was conducted to further analyze the electronic structure and optical properties.

## 2 Synthesis and experimental methods

### 2.1 Synthesis

**Scheme 1:**
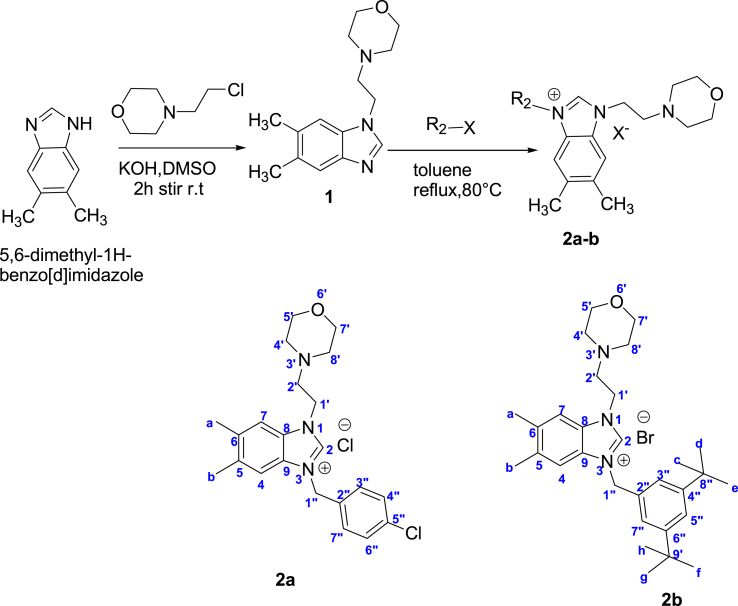
The synthetic route of compounds **2a-b**.

Benzimidazoles salts (**1**–**2**) were prepared via the two step Nalkylation process as depicted in Scheme 1.

Compound **1** was obtained by N-alkylation of 5,6-dimethylbenzimidazole by 4-(2-chloroethyl)morpholine in DMSO in the presence of KOH at room temperature for 2 h. The benzimidazolium salt **2a** were prepared by reacting compound **1**, with aryl chloride in refluxing toluene for 48 h (Scheme 1). The salt (**2b**) was synthesized by the quaternization of the intermediate (**1**) with 1.3-di-*tert*-butyl-5-chloromethyl benzene in toluene for 24 h in degassed dimethylformamide at 80 °C. The reaction has been monitored following thin layer chromatography, and after this time the formation of salts (**2**), has been observed for every target compound. The benzimidazolium salts (**2**) were air- and moisture stable both in the solid state and in solution. The FTIR spectroscopy, ^1^H- and ^13^C{1H} NMR spectroscopy, and elemental analysis data of the title compounds confirm the proposed structures. NMR spectra of all the compounds were analyzed in d-CDCl_3_. In the ^1^ H NMR spectra, acidic protons (NCHN) for benzimidazolium salts (**2**) were seen at 11.61, and 11.17 ppm, respectively, as a characteristic sharp singlet. In the 13C{1H} NMR spectra of benzimidazoles salts (**2**), the NCHN carbon were detected as typical singlets at 143.2 and 152.2 ppm, respectively. These values are consistent with related literature [20,21]. In the IR spectra, the m (CN) bands for salts (**2a-b**) were observed at 1650 and 1666 cm^−1^, respectively. The stability studies of the salts (**2a-b**) were carried out as pre reported procedure with minor modifications [59]. Dmso-d_6_ solutions of them were subjected stability tests for a period of one 2 week by ^1^H NMR. It is ensued that the salts are stable in solutions.

### 2.2 Experimental

a Synthesis of 1.

Substituted benzimidazole (1) was prepared by reacting 5,6-dimethylbenzimidazole with potassium hydroxide (4 mmol, 2.5 g). Then, the corresponding 4-(2-chloroethyl)morpholine in DMSO (3 mmol) was added slowly. After stirring for 2 h at room temperature, the mixture was refluxed for 16 h. The reaction was followed by thin layer chromatography (TLC). After cooling to room temperature, the solvent was evaporated under reduced pressure. The residue obtained was dissolved in 4 mL of dichloromethane. After filtration, the solution is concentrated under reduced pressure. The yellow solid obtained is dried under vacuum and the isolated product is characterized by NMR spectroscopy.

b Synthesis of benzimidazoles salts 2

The reaction of 1-(2-morpholinoethyl)-5,6-dimethylbenzimidazole (1 mmol) with alkyl chloride/alkyl bromide (1,04 mmol) in toluene (10 mL) at 80 °C for 48 h afford benzimidazole salts. A white solid was obtained after adding Diethyl ether (15 mL), which was subsequently filtered off. After washing with diethyl ether (3 * 15 mL) the solid was dried under vacuum.

#### 2.2.1 3-(4-chlorobenzyl)-1-(2-morpholinoethyl)-5,6-dimethylbenzimidazolium chloride (2a)

Yield: 73 %; Mp 244 °C; ν (CN) = 1563 cm^−1^; HR-AM (H-ESI II) analysis calculated *(m/z*) for cationic part of [C_22_H_27_ClN_3_O]^+^: 384.93; found *(m/z*): 384.1787.^1^H NMR (400 MHz, CDCl_3_) *δ* (ppm) = 2.38 (d, 6H, CH_3(a,b)_), 2.59 (s, 4H, H_4’,8’_), 2.93 (s, 2H, H_2’_), 3.62 (s, 4H, H_5’,7’_), 4.67 (s, 2H, H_1’_), 5.80 (s, 2H, H_1″_), 7.31 (t, 3H, H_4,3″,7″_); 7.46 (d, 3H, H_7,4″,6″_), 11.61 (s, 1H, H_2_); ^13^C NMR (100 MHz, CDCl_3_) *δ* (ppm) = 20.80 (C_b_), 20.82 (C_a_), 44.13 (C_1’_), 50.24 (C_4’,8’_), 53.44 (C_2’_), 56.09 (C_1″_), 66.86 (C_5’,7’_), 112.76 (C_4_), 113.23 (C_7_), 129.46 (C_8,9_), 129.57 (C_3″,7″_), 129.74 (C_6_),129.91 (C_5_), 131.88 (C_4″,6″_), 135.25 (C_5″_), 137.48 (C_2”_), 143.27 (C_2_).

#### 2.2.2 3-(3,5-di-*tert*-butylbenzyl)-1-(2-morpholinoethyl)-5,6-dimethylbenzimidazolium bromide (2b)

Yield: 92 %; Mp 247 °C; ν (CN) = 1564 cm^−1^; HR-AM (H-ESI II) analysis calculated *(m/z*) for cationic part of [C_30_H_44_N_3_O]^+^: 462.71; found *(m/z*): 462.3415.^1^H NMR (400 MHz, CDCl_3_) *δ* (ppm) = 1.28 (s, 18H, CH_3(c,d,e,f,g,h)_), 2.37 (s, 3H, CH_3(b)_), 2.42 (s, 3H, CH_3(a)_), 2.57 (s, 4H, H_4’,8’_), 2.93 (s, 2H, H_2’_), 3.56 (s, 4H, H_5’,7’_), 4.72 (t, 2H, H_1’_), 5.68 (s, 2H, H_1″_), 7.29 (d, 2H, H_3″,7″_); 7.36 (s, 1H, H_4_), 7.41 (s, 1H, H_5″_), 7.46 (s, 1H, H_7_), 11.17 (s, 1H, H_2_); ^13^C NMR (100 MHz, CDCl_3_) *δ* (ppm) = 20.79 (C_b_), 20.81 (C_a_), 31.50 (C_c,d,e,f,g,h_), 35.07 (C_8″,9″_), 52.14 (C_1’_), 53.44 (C_2’,4’,8″_), 65.97 (C_1″,5’,7’_), 112.86 (C_4_), 113.57 (C_7_), 122.84 (C_5″_), 123.42 (C_4″,6″_), 129.76 (C_8_),130.14 (C_9_), 131.93 (C_3″,7″_), 137.20 (C_5,6_), 142.36 (C_2”_), 152.29 (C_2_).

### 2.3 Material and methods of anticancer study

The cytotoxicity of the compounds was assessed using 3-(4,5-dimethylthiazol-2-yl)-2,5-diphenyltetrazolium bromide (MTT) assays, as described in Ref. [22].

### 2.4 Measurement experiments

The FT-IR spectrum of the title compounds diluted in the KBr pellets was recorded on a Thermo Electron Nexus 670 spectrophotometer in the range of 400–4000 cm^−1^. The ^1^H NMR and ^13^C NMR measurements were carried out using a Bruker AV400 NMR spectrometer with tetramethylsilane (TMS) as an internal standard in CDCl_3_. The UV–Vis absorption was carried out on a Thermo Nicolet Evolution 500 spectrometer in solid state. Fluorescence spectrum was recorded with an Edinburgh fluorescence lifetime spectrometer 920 in solid state, the range for recording fluorescence emission was from 330 to 600 nm, and the excitation wavelength was set at 328 nm. All these spectra were measured at room temperature.

#### 2.4.1 Computational details

Organic salt derivative geometries were thoroughly optimized using Density Functional Theory (DFT) [[23], [24], [25]]. Various exchange-correlation (xc) functionals were employed in the process, such as B3LYP [[26], [27], [28]], CAM-B3LYP [29], M06 [30],ωB97X-D [31,32], along with the inclusion of dispersion effects using the B3LYP-d3 [33] hybrid functional. Several basis sets were tested such as: 6–311++G (d,p) [34], 6–31++G (d,p) [34], cc-pvdz [35] and cc-pvtz [35]. These computations yielded valuable insights into the properties and behavior of these compounds, providing valuable guidance for experimental research. To ensure convergence, criteria were established for residual forces acting on the atoms, with a threshold set at 1.5 × 10^−5^ Hartree per Bohr. Subsequently, vibration frequencies were computed for the optimized geometries representing the ground state, and the observed frequencies confirm the validity of these geometries as energy minima on the potential energy surface.

The ground-state geometries were optimized, and then the TDDFT method [[36], [37], [38]] was used to calculate the 30 lowest electronic excitation energies (${\Delta E}_{0n}$) and oscillator strengths ($f_{0n}$). Various exchange-correlation (xc) functionals were applied, including global hybrid functionals such as B3LYP, CAM-B3LYP, M06, ωB97X-D and B3LYP-d3, all consistently using the cc-pvdz basis set. To simulate UV/visible absorption spectra, each transition was associated with a Gaussian function characterized by a full width at half maximum (FWHM) of 0.33 eV.

All calculations were carried out using Gaussian A16 program [39].

### 2.5 X-ray crystallography

Colorless crystals of **2a** and **2b** with approximate dimensions 0.54 × 0.15 × 0.06 mm^3^ and 0.59 × 0.58 × 0.46 mm^3^, respectively, were chosen for the crystallographic study and then carefully mounted on goniometer of a STOE diffractometer with an IPDS II image plate detector. All diffraction measurements were performed at room temperature (296 K) using graphite monochromated MoK*α* radiation at a wavelength of 0.71073 Å by applying the *ω*-scan method. Data collection and cell refinement were carried out using X-AREA [Stoe & Cie, X-AREA Version 1.18 and X-RED32 Version 1.04, Stoe & Cie, Darmstadt, Germany, 2002.] while data reduction was applied using X-RED32 [Stoe & Cie, X-AREA Version 1.18 and X-RED32 Version 1.04, Stoe & Cie, Darmstadt, Germany, 2002.]. The structures have been solved by SHELXT method [40], and refined by a full-matrix least squares technique on *F*^2^ with SHELXL-2019/3 [41]. All H atoms were located in difference maps and then treated as riding atoms, fixing the bond lengths at 0.93, 0.97 and 0.96 Å for aromatic CH, CH_2_ and CH_3_ atoms, respectively. The displacement parameters of the H atoms were fixed at *U*_iso_(H) = 1.2*U*_eq_ (1.5*U*_eq_ for CH_3_). CCDC 2280566 and 2280567 contains the supplementary crystallographic data for the two compounds reported in this paper.

The Hirshfeld surfaces and the associated 2D fingerprint plot were calculated using crystal Explorer 3.1 [42].

### 2.6 Crystal structure

**Fig. 2:**
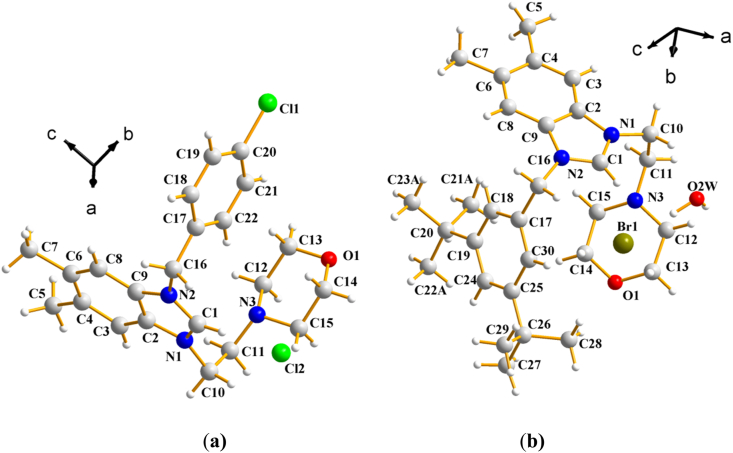
The molecular structure of salt **2a** (**a)** and **2b** (**b) (**for clarity only major sites occupancy are represented).

**Fig. 3:**
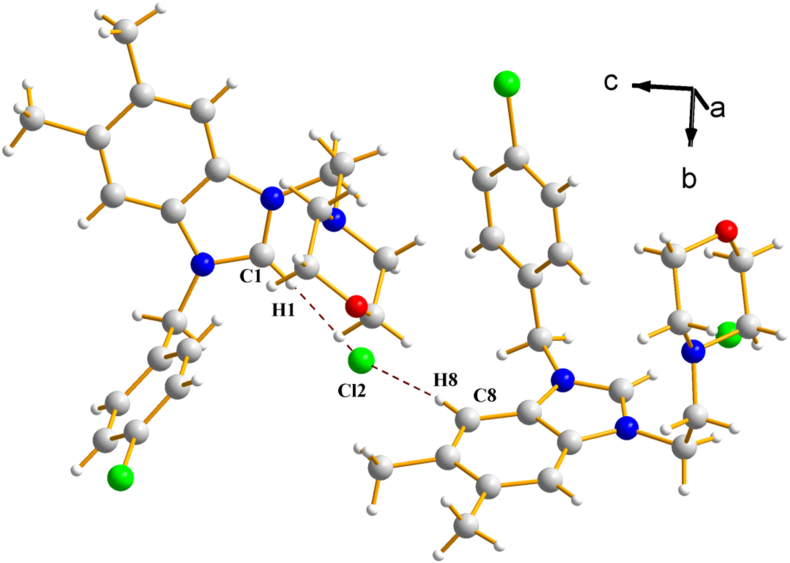
View of the crystal structure for salt **2a** showing the hydrogen bonds C–H⋅⋅⋅Cl (dashed red lines). (For interpretation of the references to color in this figure legend, the reader is referred to the Web version of this article.)

**Fig. 4:**
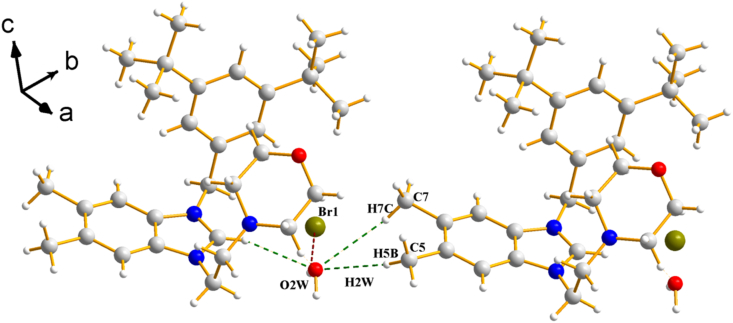
View of the crystal structure for salt **2b** showing the hydrogen bonds O–H⋅⋅⋅Br (dashed red lines) and C–H⋅⋅⋅O (dashed green lines). (For interpretation of the references to color in this figure legend, the reader is referred to the Web version of this article.)

The molecular structures of the two salts (**2a)** and **(2b)** with the adopted atom-labelling scheme were shown in Fig. 2a and b. The crystallographic data for the two studied salts **(2a)** and **(2b)** were detailed in Table 1. Single-crystal X-ray diffraction analysis revealed that salts **(2a)** and **(2b)** crystallizes in the two centrosymmetric space group *P*2_*1*_/*c* and *P -1* of monoclinic and triclinic system respectively. The crystal structure of salt **(2a)** was stabilized by weak intra and intermolecular C–H⋯Cl hydrogen bonds [43] (Fig. 3). The structure of salt **(2b**) were held together by weak intra and intermolecular O–H⋯ Br and C–H⋯O hydrogen bonds [44,45] (Fig. 4).

**Table 1:** Crystallographic data for the two studied salts **(2a)** and **(2b)**.

	(2a)	(2b)
*Chemical formula*	*C*_*22*_*H*_*27*_*ClN*_*3*_*O.Cl*	*C*_*30*_*H*_*44*_*N*_*3*_*O·0.5H*_*2*_*O·Br*
*Formula weight (g mol*^*−*^*^1^)*	*420.36*	*551.60*
*Temperature (K)*	*296*	*296*
*Crystal system*	*Monoclinic*	*Triclinic*
*Space group*	*P*2_*1*_/*c*	*P -1*
*a, b, c (Å*	*9.999 (1), 14.145 (2), 16.364 (2)*	*9.989 (1), 10.124 (1), 16.158 (2)*
*α, β, γ (°)*	*90, 104.9 (1), 90*	*90.9 (1), 101.2 (9), 106.7 (9)*
*Volume (Å*^*3*^*)*	*2236.8 (5)*	*1530.5 (3)*
*Z*	*4*	*2*
*Density calculated (mg m*^*−3*^*)*	*1.248*	*1.197*
*Absorption coefficient (mm*^*−*^*^1^)*	*0.31*	*1.37*
*F(000)*	*888*	*586*
*Diffractometer*	*STOE IPDS 2*	*STOE IPDS 2*
*Index ranges*	*−11* ≤ *h ≤ 11, −16 ≤ k ≤ 16, −18* ≤ *l ≤ 19*	*−11* ≤ *h ≤ 12, −12* ≤ *k ≤ 12, −19* ≤ *l ≤ 19*
*θ range for data collection (°)*	*1.9 ≤ θ ≤ 25.1*	*1.3* ≤ *θ ≤ 25.5*
*Collected reflections*	*15650*	*20576*
*Independent/observed reflections*	*3963/1376*	*5658, 3498*
*R*_*int.*_	*0.146*	*0.095*
*Refinement method*	*Full-matrix least-squares on F*^*2*^	*Full-matrix least-squares on F*^*2*^
*Goodness-of-fit on F*^*2*^	*0.86*	*0.97*
*Final R indices [F*^*2*^ > *2σ(F*^*2*^*)]*	*R = 0.057,* w*R = 0.123*	*R = 0.057,* w*R = 0.163*
*Δρ*_*max.*_*, Δρ*_*min.*_*(e/Å*^*3*^*)*	*0.22–0.16*	*0.55–0.62*

**Fig. 5:**
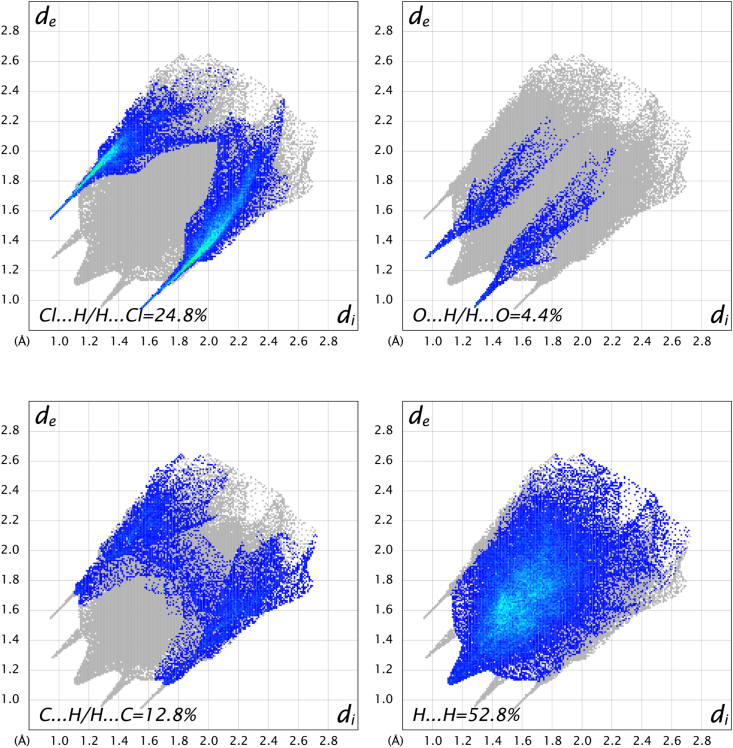
2D Fingerprint plots of salt **2a**.

**Fig. 6:**
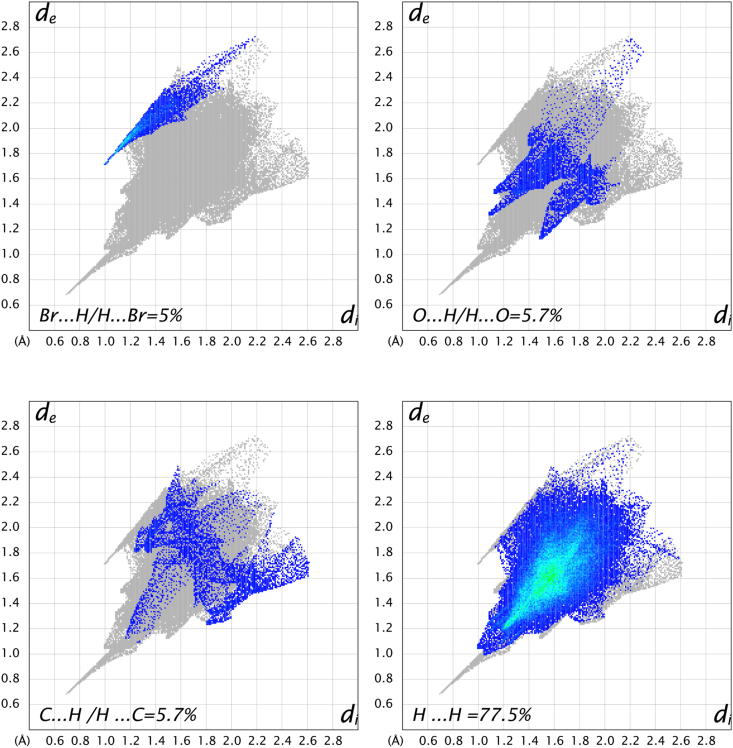
2D Fingerprint plots of salt **2b**.

In order to study the weak and van der Waals interactions, 2D fingerprint plots of the calculated Hirshfeld surface for the two salts were evaluated and revealed that the structure of salt **2a** was dominated by H⋯H (52.8 %), Cl⋯H (24.8.6 %), C⋯H (12.8 %) and O⋯H (4.4 %) van der Waals interactions (Fig. 5). In the other hand the structure of salt **2b** was dominated by H⋯H (57.5 %), Br⋯H (5 %), C⋯H (5.7 %) and O⋯H (5.7 %) van der Waals interactions (Fig. 6). The difference in the contribution of these interactions between the two structures can be explained by the position of halogen atoms.

#### 2.6.1 Structural study

The selection of the suitable functional to optimize the organic salt geometries was based on Koopmans approach [46], which states that the electron affinity and ionization energy should equal two by two to the absolute values of HOMO and LUMO, respectively. A parameter Δ was proposed in order to compare the performance of each functional. Δ is expressed as follows:

$$\Delta =|\text{AE}+\varepsilon_{\text{HOMO}}\left|+\right|\text{IE}+\varepsilon_{\text{LUMO}}|$$

According to the data in Table 2, the ωb97x-d functional showed the lowest Δ value, and consequently, it should be considered the best functional. ωb97x-d is recommended for salt molecules and intramolecular interactions [47]. Four basis-sets were tested and a comparative structural study was conducted. The experimental and computed bond length values of Salt1 (**2a**) are summarized in Table 3 cc-pvdz is the suitable basis-set since it exhibited the lowest RMSD value with high correlation factor. We proceeded to perform the optimization of salts1 (**2a**) and **(2b)** using the ωB97X-D/cc-pVDZ level of theory, as it demonstrated a highly favorable correlation with experimental data (with an RMSD of 0) as indicated in Table 4.

**Table 2:** Computed energy values at neutral (E(N)), cationic (E (N-1)), and anionic (E (N+1)) states in atomic units (a.u) and electronic structure properties (HOMO ($\varepsilon_{\text{HOMO}}$), LUMO ($\varepsilon_{\text{LUMO}}$), ionization energy (IE), electronic affinity (AE) in (eV) for **2a** using several xc functionals with 6–311++G (d,p) basis set.

	E(N)	E (N-1)	E (N+1)	AE	IE	$\varepsilon_{\text{HOMO}}$	$\varepsilon_{\text{LUMO}}$	Δ
**B3LYP**	−2014.827135	−2014.552226	−2014.848875	7.48	0.59	−5.43	−1.94	3.39
**ωb97X-D**	−2014.454475	−2014.163326	−2014.465784	7.92	0.31	−7.64	−0.08	0.51
**M06**	−2013.954407	−2013.673113	−2013.977851	7.65	0.64	−5.81	−1.39	2.59
**B3LYP-D3**	−2014.898062	−2014.623232	−2014.918071	7.48	0.54	−5.52	−1.93	3.34
**CAM-B3LYP**	−2014.237815	−2013.944326	−2014.253447	7.99	0.43	−7.17	−0.76	1.15

**Table 3:** Experimental geometric data and computed bond length, RMSD in (Å) and correlation factor (r^2^) values of salt1 (**2a**) at ωb97x-d level with different basis sets.

**Bond length**	**EXP**	**6**–**311g** + + **(d,p)**	**6**–**31g** + + **(d,p)**	**cc-pvdz**	**cc-pvtz**	
**N1–C1**	1.331	1.334	1.337	1.335	1.328	
**N2–C1**	1.348	1.339	1.342	1.332	1.326	
**C6–C8**	1372	1.394	1.397	1.392	1.384	
**C19–C20**	1.375	1.390	1.397	1.393	1.386	
**C20–C21**	1.375	1.393	1.402	1.391	1.383	
**C17–C22**	1.376	1.399	1.403	1.398	1.389	
**C2–C9**	1.385	1.400	1.397	1.396	1.389	
**C18–C19**	1.387	1.394	1.399	1.392	1.384	
**C17–C18**	1.387	1.395	1.394	1.398	1.390	
**C21–C22**	1.388	1.391	1.394	1.394	1.386	
**N1–C2**	1.395	1.397	1.397	1.393	1.388	
**C8–C9**	1.397	1.392	1.395	1.394	1.386	
**C2–C3**	1.398	1.392	1.396	1.393	1.385	
**C3–C4**	1.399	1.394	1.397	1.392	1.384	
**N2–C9**	1.401	1.399	1.400	1.393	1.388	
**C14–O1**	1.404	1.429	1.431	1.417	1.415	
**C4–C6**	1.413	1.424	1.427	1.424	1.416	
**C13–O1**	1.419	1.417	1.419	1.407	1.405	
**N3–C12**	1.441	1.466	1.467	1.456	1.453	
**N3–C15**	1.461	1.474	1.475	1.462	1.459	
**N3–C11**	1.462	1.454	1.455	1.443	1.440	
**N1–C10**	1.469	1.470	1.470	1.454	1.452	
**N2–C16**	1.469	1.471	1.470	1.471	1.466	
**C14–C15**	1.501	1.522	1.524	1.521	1.517	
**C12–C13**	1.504	1.526	1.527	1.522	1.518	
**C10–C11**	1.516	1.534	1.536	1.536	1.532	
**C16–C17**	1.520	1.515	1.402	1.512	1.507	
**C4–C5**	1.529	1.509	1.511	1.506	1.503	
**C6–C7**	1.530	1.509	1.511	1.506	1.503	
**C20–Cl1**	1.743	1.756	1.756	1.749	1.741	
	–	0.987	0.945	0.985	0.987	**r**^**2**^
	–	0.024	0.033	0.010	0.012	**RMSD**

**Table 4:** Experimental geometric data and computed bond length, RMSD in (Å) and coorelation factor values of salt2 (**2b**) at different methods.

Site	Experiment	B3LYP/6–311++G (d,p)	ωb97x-d/6–311++G (d,p)	ωb97x-d/cc-pvdz
**C2–C3**	1.393	1.393	1.390	1.395
**C3–C4**	1.391	1.391	1.385	1.389
**C2–N1**	1.398	1.398	1.392	1.393
**C2–C9**	1.401	1.399	1.391	1.395
**C4–C5**	1.509	1.509	1.506	1.507
**C4–C6**	1.428	1.428	1.424	1.428
**C1–N1**	1.330	1.332	1.325	1.327
**N1–C10**	1.481	1.483	1.473	1.472
**N2–C9**	1.397	1.397	1.391	1.393
**C8–C9**	1.395	1.395	1.392	1.397
**C6–C8**	1.392	1.393	1.387	1.391
**C6–C7**	1.508	1.509	1.505	1.506
**C1–N2**	1.344	1.344	1.336	1.339
**C10–C11**	1.525	1.521	1.518	1.516
**N2–C16**	1.477	1.478	1.466	1.465
**N3–C11**	1.471	1.470	1.462	1.461
**C16–C17**	1.513	1.510	1.507	1.509
**N3–C12**	1.472	1.473	1.460	1.460
**N3–C15**	1.470	1.468	1.455	1.455
**C17–C18**	1.401	1.401	1.397	1.401
**C17–C30**	1.389	1.390	1.384	1.389
**C12–C13**	1.523	1.523	1.518	1.518
**C14–C15**	1.523	1.525	1.521	1.522
**C18–C19**	1.392	1.393	1.387	1.392
**C25–C30**	1.405	1.405	1.401	1.405
**O1–C13**	1.423	1.425	1.415	1.416
**O1–C14**	1.422	1.421	1.413	1.412
**C19–C20**	1.540	1.540	1.532	1.535
**C19–C24**	1.408	1.407	1.402	1.406
**C24–C25**	1.394	1.395	1.388	1.393
**C25–C26**	1.540	1.540	1.532	1.535
**C20–C21**	1.547	1.547	1.539	1.540
**C20–C22**	1.547	1.547	1.540	1.540
**C20–C23**	1.539	1.540	1.533	1.534
**C26–C27**	1.539	1.540	1.533	1.534
**C26–C28**	1.547	1.546	1.540	1.541
**C26–C29**	1.547	1.547	1.539	1.540
**RMSD**	–	0.000	0.040	0.020

**Fig. 7:**
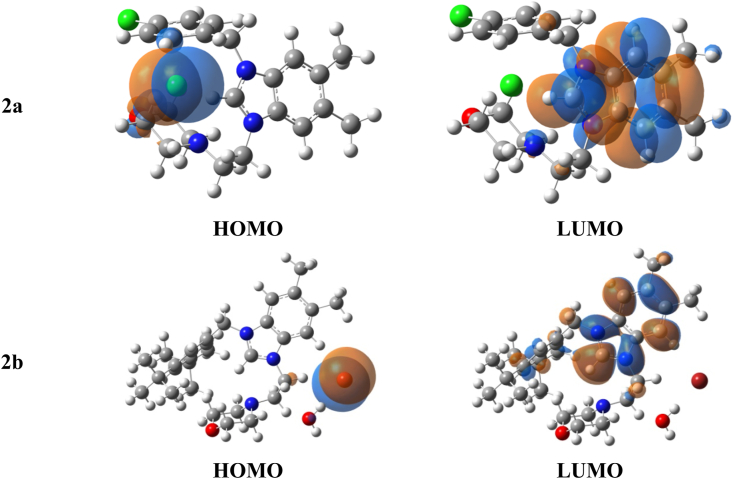
Computed HOMO and LUMO localizations of salts **2a** and **2b** at ωb97x-d/cc-pvdz level of theory.

**Fig. 8:**
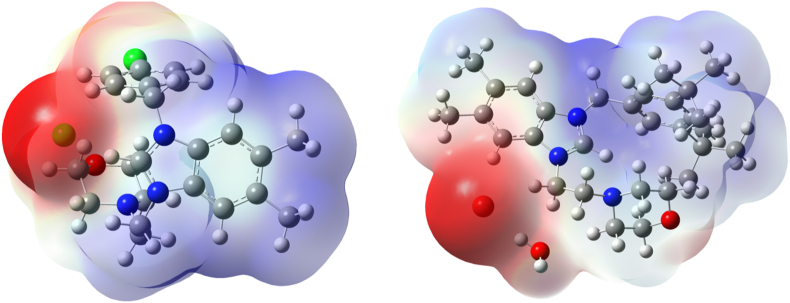
Computed MESP surfaces of Salts 1 (**2a**) and 2 (**2b**) at ωb97x-d/cc-pvdz level of theory.

A comparative structural study of salts 1 and 2 were conducted. According to the data in Table 5, a stabilization of HOMO level was observed in the case of the bromide salt (Salt2) with decreasing in electronic energy gap value by 0.93eV (approximately 1eV) comparing with chloride salt (salt1). In both salts, the LUMO is displaced to the aromatic rings, whereas the HOMO is mainly localized on bromide and chloride as shown in (Fig. 7). Hence, a thorough analysis of the three-dimensional Molecular Electrostatic Potential (MESP) and its gradient plots is vital for gaining insight into the interaction between the electronic surfaces of organic molecules and the active site of a protein, for example. MESP analysis offers valuable insights into the surface characteristics of organic materials as ligands, which play a pivotal role in non-covalent interactions with amino acid residues within protein targets. Color-coded MESP plots can be generated to visually represent the electrostatic surface of organic salts, ultimately aiding in the design of drugs that effectively complement specific targets [48]. The red-colored region, indicating nucleophilic or donor sites, is mainly concentrated on halogen atoms. In contrast, the electrophilic blue region is localized across the six-membered and five-membered rings, as illustrated in (Fig. 8). The significant reactivity of halogens in both salts has been verified through their notably high absolute (${\text{Vs}}_{\min}$) values, as presented in Table 6.

**Table 5:** Computed HOMO, LUMO and electronic energy gap values of Salt1 (**2a**) and 2 (**2b**) at ωb97-xd/cc-pvdz level of theory.

	$\varepsilon_{\text{HOMO}}$	$\varepsilon_{\text{LUMO}}$	$\Delta_{L-H}$
**2a**	−7.64	−0.08	7.56
**2b**	−6.45	0.18	6.63

**Table 6:** Computed maximum/minimum potential (${Vs}_{\max}/{Vs}_{\min}$) values in (kJ.mol^−1^) of Salts 1 (**2a**) and 2 (**2b**) at ωb97x-d/cc-pvdz level of theory.

Salt1 (2a)		Salt2 (2b)	
Site	${\text{Vs}}_{\max}/{\text{Vs}}_{\min}$	Site	${\text{Vs}}_{\max}/{\text{Vs}}_{\min}$
**N1**	42.43	Br1	−241.19
**N2**	39.71	C1	84.94
**N3**	44.85	N1	50.20
**O1**	−133.55	N2	85.10
**Cl1**	−53.67	N3	63.08
**Cl2**	−224.17	O1	−85.65

### 2.7 FTIR spectra

**Fig. 9:**
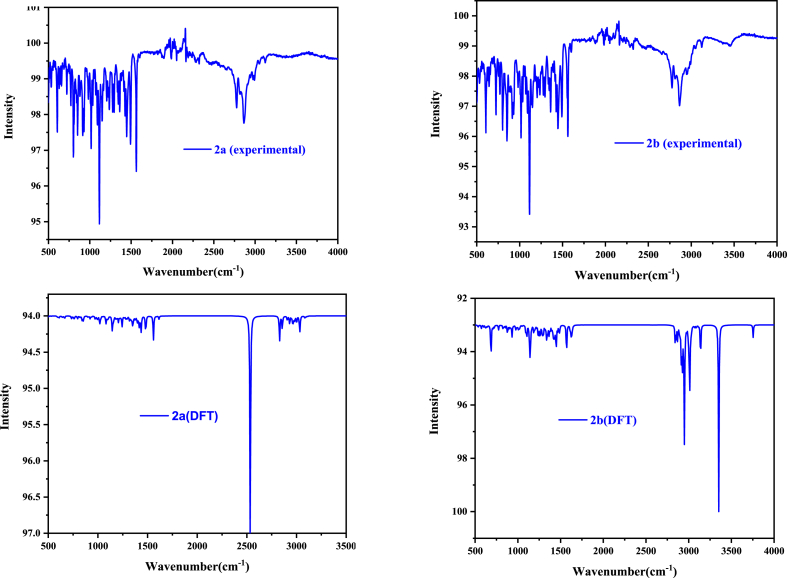
Experimental and DFT FTIR spectra of Salts 1 (**2a**) and 2 (**2b**).

The Infrared spectrum of the synthesized compound was recorded with a PerkinElmer Spectrum 100 Gladi ATR FT/IR spectrophotometer in the range of 4000–400 cm^−1^. The experimental and calculated infrared spectra of the **2a** and **2b** salts are shown in Fig. 9. The broad bands observed in the range of 2850–3200 cm^−1^ were assigned to the = C–H stretching of the aromatic ring and –C–H stretching of the alkyl group. Theoretically, this stretching is found in the range of 2800–3090 cm^−1^.

The bands observed at 1560 and 1440 cm^−1^ were attributed to the C=N stretching and (CH_2_) binding. Theoretically, these vibration modes were found at 1580 cm^−1^ and 1320 cm^−1^, respectively. The most intense band is observed at 1160 cm^−1^ and it may be assigned to C–O stretching of C–*O*–C ether binding and it's confirmed theoretically.

### 2.8 Optical properties

**Fig. 10:**
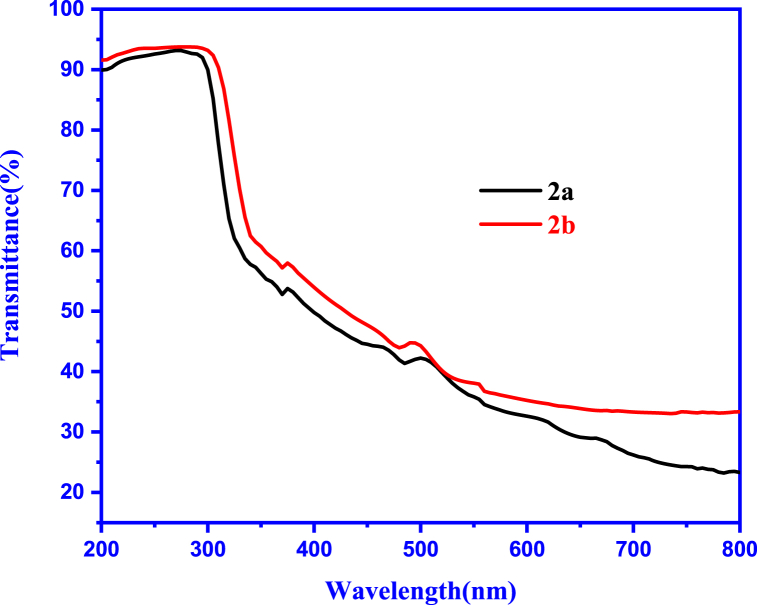
Experimental UV-absorption spectra of **2a** and **2b** salts.

UV–visible and photoluminescence (PL) measurements were conducted in the solid state at room temperature using a UV–Vis spectrometer (PerkinElmer lambda 950) and both spectra of **2a** and **2b** are sketched in Fig. 10. A prominent absorption peak was observed at 265 nm and the obtained value is close to other benzimidazole derivatives as anti-cancer agents [49]. To determine the optical energy gap, a 10 % absorption UV spectrum was simulated using an approach that identifies the intersection of UVabs and UVabs (scaled by 10 %) as an effective method [50]. As a result, we found the optical energy gap for both salts to be 4.51 eV, with an intersection wavelength value of 275 nm. This underscores the suitability of the ωb97x-d functional for optimizing these chloride and bromide salts, as it consistently yields higher electronic bandgap values compared to optical ones.

**Fig. 11:**
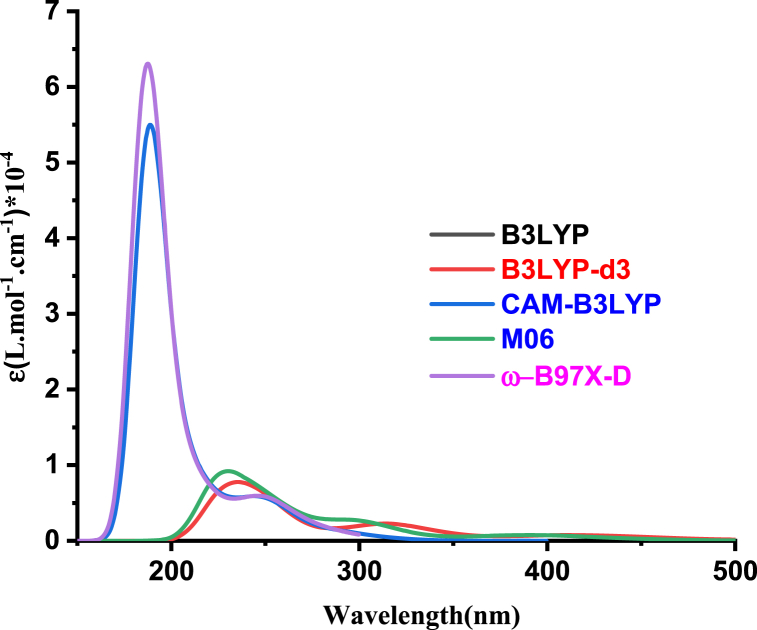
Computed UV-abs spectra of Salt1 (**2a**) at cc-pvdz basis-set with different xc functionals.

**Fig. 12:**
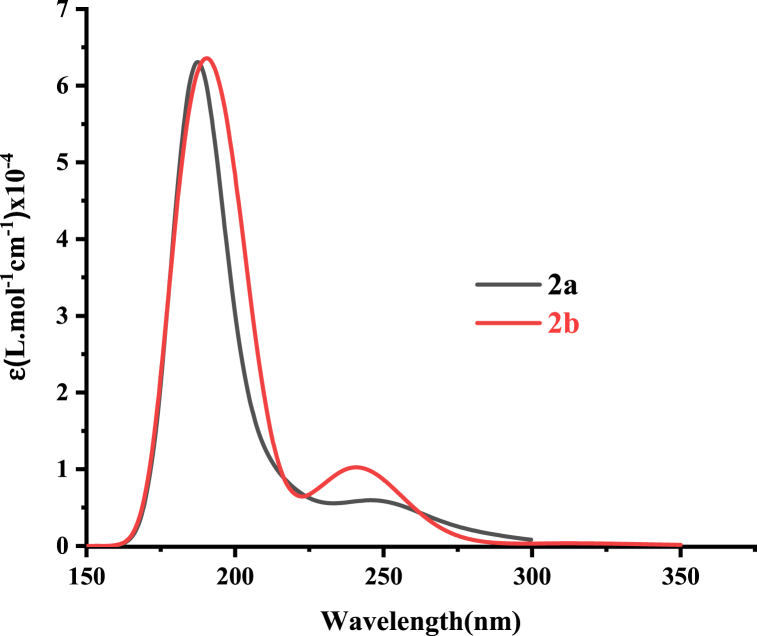
Computed UV-abs spectra of Salt1 (**2a**) and Salt2 (**2b**) at ωb97x-d/cc-pvdz level of theory.

Fig. 11 illustrates the computed TD-DFT spectra for **2a**, employing a cc-pvdz basis set with various exchange-correlation (xc) functionals. Additionally, Fig. 12 displays the calculated UV spectra for both Salts **2a** and **2b**, using the ωb97x-d/cc-pvdz level of theory. The optical parameters for both salts are summarized in Table 7.

**Table 7:** Computed TD-DFT wavelenghth ($\lambda_{TD-\text{DFT}}$) at (nm), vertical transition energy (${\Delta E}_{0-0}$) in (eV), major molecular orbital contributions (MOs) and oscillator strength (f) at ωb97x-d/cc-pvdz level of theory.

Compound	$\lambda_{\text{DFT}}$	${\Delta E}_{0-0}$	MOs	f
**Salt (2a)**	250	4.95	H (-3)→L (56 %)	0.12
	187	6.53	H-6→L (13 %)	1.12
			H-3→L+3 (12 %)	
			H-3→L+2 (10 %)	
			H-8→L+3 (10 %)	
		6.68	H-3→L+3 (25 %)	
			H-6→L+1 (24 %)	
		6.75	H-3→L+4 (23 %)	
**Salt (2b)**	241	5.02	H-4→L (57 %)	0.22
		5.32	H-5→L (76 %)	
	190	6.23	H-3→L+1 (53 %)	1.19
		6.26	H-3→L+1 (34 %)	
		6.77	H-7→L+2 (29 %)	

The calculations using ωb97x-d and cam-b3lyp functionals produced similar UV spectra, revealing two prominent absorption bands at 187 nm and 250 nm. The first band is attributed to π-π* transitions, while the second one may be associated with a charge transfer phenomenon occurring between chloride and the benzimidazole derivative.

Based on the spectra presented in Fig. 12 and the data in Table 7, a slight blue-shift and hyperchromic effect were observed in the case of the bromide salt (**2b**).

**Fig. 13:**
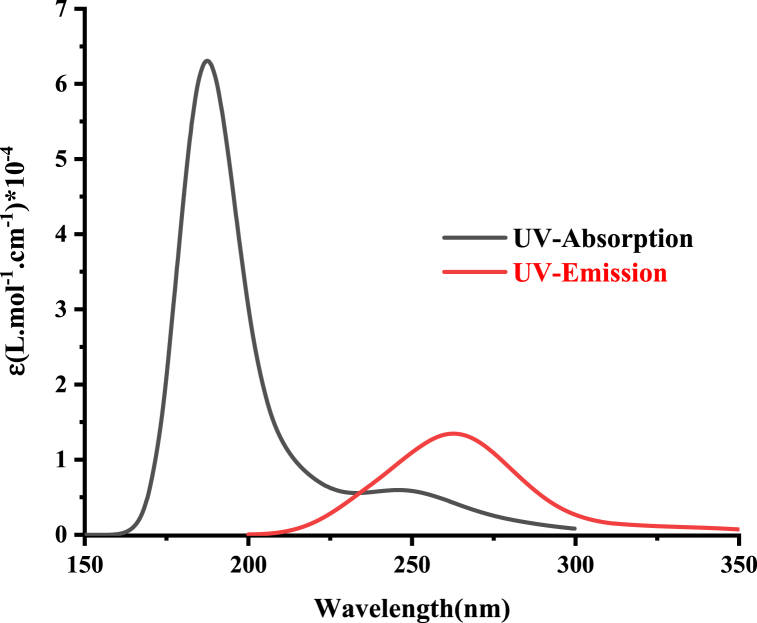
Computed UV absorption and emission spectra of **2a** at ωb97x-d/cc-pvdz level of theory.

**Fig. 14:**
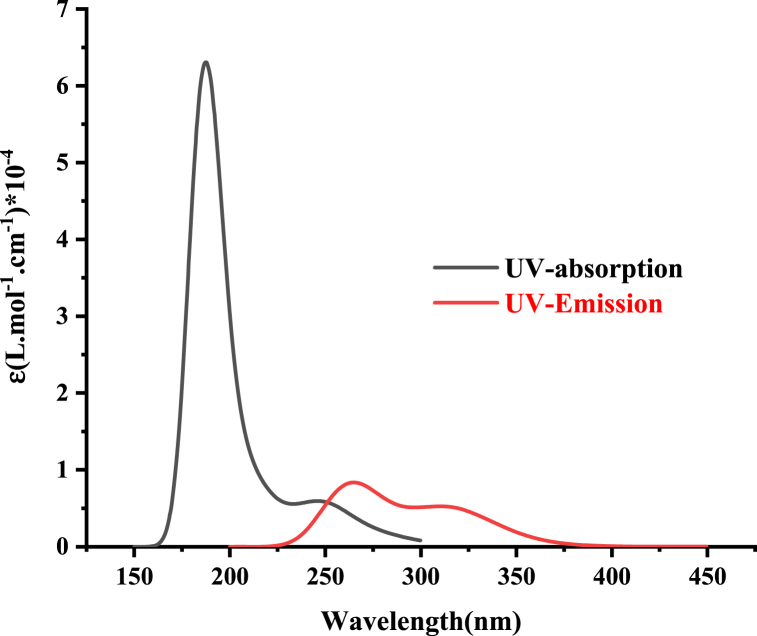
Computed UV absorption and emission spectra of **2b** at ωb97x-d/cc-pvdz level of theory.

Emission spectra for both salts are illustrated in Fig. 13 and Fig. 14, and the optical energy gap values, determined through the intersection of absorption and emission spectra, are summarized in Table 8. The optical energy gap ($E_{g\left(\text{opt}\right)}$) values are close and align well with the theoretical ones. The strong agreement between experimental and time-dependent density functional theory (TDDFT) results allows us to conclude that the selected computational approach (ωB97X-D) is reliable for investigating the excited states of these chloride and bromide salts.

**Table 8:** Computed intersection wavelength $\lambda_{\mathrm{int}}$ in (nm) and optical energy gap $E_{g\left(\text{opt}\right)}$ in (eV) of **2a** and **2b** at ωb97x-d/cc-pvdz level of theory.

Salt	$\lambda_{\mathrm{int}}$	$E_{g\left(\mathrm{opt}\right)}$
**2a**	233	5.32
**2b**	254	4.88

## 3 Evaluation of anticancer activity

MTT assay is done to measure the toxicity of a drug that interferes with the cellular viability. The well-known cytotoxic drugs targets and interfere with the reproducibility of cells to control their growth and proliferation. In short, the toxicity of drugs or any treatment is inversely proportional to the cell viability. Here, the toxicity of the samples is proved to be concentration dependent as it decreases the viability of cells along with increasing drug concentrations. No observable toxicity was seen at concentrations below 200 μL/ml of the sample as these compounds may be opted out from the treated cells by the cells own defense mechanism. The percentage of cell viability of treated cells indicate that some of the drugs can interfere with their reproducibility in a distinct way. The IC_50_ values for all compounds **1**–**2** were calculated and compared with the anticancer drug Cisplatin. All of these compounds exhibited cytotoxic effects against all cell lines at lower doses compared to cisplatin. The obtained results are presented in Table 9. All synthesized compounds showed higher cytotoxic activity. Among the synthesized compounds, compound 1 showed the lowest cytotoxic activity against all cell lines, and salt **2b** showed the highest cytotoxic activity. On the other hand, these compounds have lower cytotoxic activity for the healthy cell line. Salt **2a** showed relative significant higher cytotoxicity (165.02) in *MDA-MB-231* cancer cell line, and higher cytotoxic effect (225.8) in *HF* cells compared to cisplatin. Elaborated investigation should be conducted in this track to fulfil the need of achieving potent cytotoxic properties of the drug which can specifically reduce the cancer cell reproducibility according to previous works [51].

**Table 9:** The IC_50_ calues of compounds 1-2.

COMPOUND	*MDA-MB-231*^*A*^	*MCF-7*^*A*^	*HCT116*^*A*^	*HT-29*^*A*^
1	205.74 ± 6.79	231.38 ± 4.98	375.41 ± 2.16	215.92 ± 12.70
2A	165.02 ± 8.86	175.02 ± 6.30	219.37 ± 5.39	225.83 ± 7.72
2B	28.80 ± 0.26	28.98 ± 0.49	228.63 ± 7.13	61.28 ± 6.38
DMSO	ineffective	ineffective	ineffective	ineffective
*CIS*PLATIN^B^	13.290	11.280	14.320	0.012

IC_50_ values presented as mean ± SD of three independent experiments.

^a^: Cell lines.

^b^: Reference Drug.

## 4 Conclusion

In summary, our study aimed to assess the cytotoxicity of salts 2 compared to cisplatin against various cancer cell lines. We synthesized two benzimidazole salts and characterized their structures using FT-IR, UV, and NMR spectroscopy. Theoretical methods, specifically DFT/ωb97x-d with cc-pvdz basis sets, were employed to investigate optimized geometric parameters and vibrational frequencies, yielding good agreement between theoretical and experimental results. Furthermore, we examined the optical properties deduced from absorption UV spectra theoretically and experimentally, utilizing the TD-DFT method to compute absorption and emission spectra. Experimental findings revealed strong UV absorption at 275 nm for both 2a and 2b salts, consistent with theoretical predictions. Our findings demonstrated that both salts exhibited significant cytotoxic activity against multiple cell lines, including Furthermore, The in vitro anticancer activities of these compounds were evaluated against *MDA-MB-231*, *MCF-7*, *HT-29* and healthy cell line (*HF*). These results suggest the potential of salts **2** as novel candidates for further investigation in cancer therapy.

## Data availability statement

The data that support the finding of this study are available on request from the corresponding author.

## Author declaration

We wish to confirm that there are no known conflicts of interest associated with this publication and there has been no significant financial support for this work that could have influenced its outcome.

## CRediT authorship contribution statement

**Mohamed Oussama Zouaghi:** Data curation. **Donia Bensalah:** Conceptualization. **Sabri Hassen:** Conceptualization. **Youssef Arfaoui:** Conceptualization. **Lamjed Mansour:** Formal analysis. **Namık Özdemir:** Data curation. **Hakan Bülbül:** Conceptualization. **Nevin Gurbuz:** Conceptualization. **Ismail Özdemir:** Conceptualization. **Naceur Hamdi:** Project administration.

## Declaration of competing interest

The authors declare that they have no known competing financial interests or personal relationships that could have appeared to influence the work reported in this paper.

## References

[1] Bansal Y., Silakari O. (2012). The therapeutic journey of benzimidazoles: a review. Bioorg. Med. Chem..

[2] Demirci S., Basoglu S., Bozdereci A., Demirbas N. (2013). Preparation and antimicrobial activity evaluation of some new bi- and triheterocyclic azoles. Med. Chem. Res..

[3] Thamban Chandrika N., Shrestha S.K., Ranjan N., Sharma A., Arya D.P., Garneau-Tsodikova S. (2018). New application of neomycin B–bisbenzimidazole hybrids as antifungal agents. ACS Infect. Dis..

[4] Starčević K., Kralj M., Ester K., Sabol I., Grce M., Pavelić K., Karminski-Zamola G. (2007). Synthesis, antiviral and antitumor activity of 2-substituted-5-amidino-benzimidazoles. Bioorg. Med. Chem..

[5] Ito Y., Shibata K., Hongo A., Kinoshita M. (1998). Ecabet sodium, a locally acting antiulcer drug, inhibits urease activity of Helicobacter pylori. Eur. J. Pharmacol..

[6] Gellis A., Kovacic H., Boufatah N., Vanelle P. (2008). Synthesis and cytotoxicity evaluation of some benzimidazole-4,7-diones as bioreductive anticancer agents. Eur. J. Med. Chem..

[7] Kucuk C. (2023). Synthesis, characterization, DFT studies, and molecular docking investigation of silver nitrate complex of 5-benzimidazole carboxylic acid as targeted anticancer agents. J. Mol. Struct..

[8] Youssif B.G.M., Morcoss M.M., Bräse S., Abdel-Aziz M., Abdel-Rahman H.M., Abou El-Ella D.A., Abdelhafez E.S.M.N. (2024). Benzimidazole-based derivatives as apoptotic antiproliferative agents: design, synthesis, docking, and mechanistic studies. Molecules.

[9] Sethi P., Bansal Y., Bansal G. (2018). Synthesis and PASS-assisted evaluation of coumarin–benzimidazole derivatives as potential anti-inflammatory and anthelmintic agents. Med. Chem. Res..

[10] Boiani M., Gonzalez M. (2005). Imidazole and benzimidazole derivatives as chemotherapeutic agents. MRMC.

[11] Xiong J.-F., Li J.-X., Mo G.-Z., Huo J.-P., Liu J.-Y., Chen X.-Y., Wang Z.-Y. (2014). Benzimidazole derivatives: selective fluorescent chemosensors for the picogram detection of picric acid. J. Org. Chem..

[12] Atar M., Taspinar Ö., Hanft S., Goldfuss B., Schmalz H.-G., Griesbeck A.G. (2019). Hydrogen peroxide sensors based on fluorescence quenching of the 2-AminobenzimidazoleFluorophore. J. Org. Chem..

[13] Desiraju G.R. (1995). Supramolecular synthons in crystal engineering—a new organic synthesis. Angew. Chem., Int. Ed. Engl..

[14] Otmacic Curkovic H., Stupnisek-Lisac E., Takenouti H. (2010). The influence of pH value on the efficiency of imidazole based corrosion inhibitors of copper. Corrosion Sci..

[15] Madkour L.H., Elshamy I.H. (2016). Experimental and computational studies on the inhibition performances of benzimidazole and its derivatives for the corrosion of copper in nitric acid. Int J Ind Chem.

[16] Benavent L., Baeza A., Freckleton M. (2018). Chiral 2-aminobenzimidazole as bifunctional catalyst in the asymmetric electrophilic amination of unprotected 3-substituted oxindoles. Molecules.

[17] Arjmand F., Parveen S., Afzal Mohd, Shahid Mohd (2012). Synthesis, characterization, biological studies (DNA binding, cleavage, antibacterial and topoisomerase I) and molecular docking of copper(II) benzimidazole complexes. J. Photochem. Photobiol. B Biol..

[18] López-Rodríguez M.L., Benhamú B., Morcillo M.J., Tejada I.D., Orensanz L., Alfaro M.J., Martín M.I. (1999). Benzimidazole derivatives. 2. Synthesis and Structure−Activity relationships of new azabicyclic benzimidazole-4-carboxylic acid derivatives with affinity for serotoninergic 5-HT _3_ receptors. J. Med. Chem..

[19] Mahmood K., Hashmi W., Ismail H., Mirza B., Twamley B., Akhter Z., Rozas I., Baker R.J. (2019). Synthesis, DNA binding and antibacterial activity of metal(II) complexes of a benzimidazole Schiff base. Polyhedron.

[20] Szafran M., Katrusiak A., Dega-Szafran Z., Kowalczyk I. (2011). The structure of 4-(trimethylammonium)benzoic acid chloride studied by X-ray diffraction, DFT calculations, NMR and FTIR spectroscopy. J. Mol. Struct..

[21] Güllüoğlu M.T., Erdogdu Y., Karpagam J., Sundaraganesan N., Yurdakul Ş. (2011). DFT, FT-Raman, FT-IR and FT-NMR studies of 4-phenylimidazole. J. Mol. Struct..

[22] Sharma N., Arya G., Kumari R., Gupta N., Nimesh S. (2019). Evaluation of anticancer activity of silver nanoparticles on the A549 human lung carcinoma cell lines through alamar blue assay. BIO-PROTOCOL.

[23] Hohenberg P., Kohn W. (1964). Inhomogeneous electron gas. Phys. Rev..

[24] Kohn W., Sham L.J. (1965). Self-consistent equations including exchange and correlation effects. Phys. Rev..

[25] Zouaghi M.O., Amri N., Hassen S., Arfaoui Y., Özdemir N., Özdemir I., Hamdi N. (2023). Biological determination, molecular docking and Hirshfeld surface analysis of rhoduim(I)-N-heterocyclic carbene complex: synthesis, crystal structure, DFT calculations, optical and non linear optical properties. Inorg. Chim. Acta..

[26] Becke A.D. (1993). Density-functional thermochemistry. III. The role of exact exchange. J. Chem. Phys..

[27] Stephens P.J., Devlin F.J., Chabalowski C.F., Frisch M.J. (1994). Ab initio calculation of vibrational absorption and circular dichroism spectra using density functional force fields. J. Phys. Chem..

[28] Zouaghi M.O., Arfaoui Y., Champagne B. (2021). Density functional theory investigation of the electronic and optical properties of metallo-phthalocyanine derivatives. Opt. Mater..

[29] Yanai T., Tew D.P., Handy N.C. (2004). A new hybrid exchange–correlation functional using the Coulomb-attenuating method (CAM-B3LYP). Chem. Phys. Lett..

[30] Zhao Y., Truhlar D.G. (2008). The M06 suite of density functionals for main group thermochemistry, thermochemical kinetics, noncovalent interactions, excited states, and transition elements: two new functionals and systematic testing of four M06-class functionals and 12 other functionals. Theor. Chem. Acc..

[31] Chai J.-D., Head-Gordon M. (2008). Long-range corrected hybrid density functionals with damped atom–atom dispersion corrections. Phys. Chem. Chem. Phys..

[32] Chai J.-D., Head-Gordon M. (2008). Systematic optimization of long-range corrected hybrid density functionals. J. Chem. Phys..

[33] Grimme S., Antony J., Ehrlich S., Krieg H. (2010). A consistent and accurate *ab initio* parametrization of density functional dispersion correction (DFT-D) for the 94 elements H-Pu. J. Chem. Phys..

[34] Curtiss L.A., McGrath M.P., Blaudeau J.-P., Davis N.E., Binning R.C., Radom L. (1995). Extension of Gaussian-2 theory to molecules containing third-row atoms Ga–Kr. J. Chem. Phys..

[35] Wilson A.K., van Mourik T., Dunning T.H. (1996). Gaussian basis sets for use in correlated molecular calculations. VI. Sextuple zeta correlation consistent basis sets for boron through neon. J. Mol. Struct.: THEOCHEM.

[36] Casida M.E. (1995). Recent Advances in Computational Chemistry.

[37] Bauernschmitt R., Ahlrichs R. (1996). Treatment of electronic excitations within the adiabatic approximation of time dependent density functional theory. Chem. Phys. Lett..

[38] Tozer D.J., Handy N.C. (1998). Improving virtual Kohn–Sham orbitals and eigenvalues: application to excitation energies and static polarizabilities. J. Chem. Phys..

[39] Gaussian 16, Revision B.01 M.J. Frisch, G.W. Trucks, H.B. Schlegel, G.E. Scuseria, M.A. Robb, J.R. Cheeseman, G. Scalmani, V. Barone, G.A. Petersson, H. Nakatsuji, X. Li, M. Caricato, A.V. Marenich, J. Bloino, B.G. Janesko, R. Gomperts, B. Mennucci, H.P. Hratchian, J.V. Ortiz, A.F. Izmaylov, J.L. Sonnenberg, D. Williams-Young, F. Ding, F. Lipparini, F. Egidi, J. Goings, B. Peng, A. Petrone, T. Henderson, D. Ranasinghe, V.G. Zakrzewski, J. Gao, N. Rega, G. Zheng, W. Liang, M. Hada, M. Ehara, K. Toyota, R. Fukuda, J. Hasegawa, M. Ishida, T. Nakajima, Y. Honda, O. Kitao, H. Nakai, T. Vreven, K. Throssell, J. A. Montgomery Jr., J.E. Peralta, F. Ogliaro, M.J. Bearpark, J.J. Heyd, E. N. Brothers, K.N. Kudin, V.N. Staroverov, T.A. Keith, R. Kobayashi, J. Normand, K. Raghavachari, A.P. Rendell, J.C. Burant, S.S. Iyengar, J. Tomasi, M. Cossi, J. M. Millam, M. Klene, C. Adamo, R. Cammi, J.W. Ochterski, R.L. Martin, K. Morokuma, O. Farkas, J.B. Foresman, D.J. Fox, Gaussian, Inc., Wallingford CT, 2016, (n.d.).

[40] Sheldrick G.M. (2015). *Shelxt* – integrated space-group and crystal-structure determination. Acta Crystallogr A Found Adv.

[41] Sheldrick G.M. (2015). Crystal structure refinement with *SHELXL*. Acta Crystallogr C Struct Chem.

[42] Spackman M.A., Byrom P.G. (1997). A novel definition of a molecule in a crystal. Chem. Phys. Lett..

[43] Lu B.-Y., Li Z.-M., Zhu Y.-Y., Zhao X., Li Z.-T. (2012). Assessment of the intramolecular C–H⋯X (X=F, Cl, Br) hydrogen bonding of 1,4-diphenyl-1,2,3-triazoles. Tetrahedron.

[44] Sandoval-Lira J., Fuentes L., Quintero L., Höpfl H., Hernández-Pérez J.M., Terán J.L., Sartillo-Piscil F. (2015). The stabilizing role of the intramolecular C–H···O hydrogen bond in cyclic amides derived from α-methylbenzylamine. J. Org. Chem..

[45] Riley K.E., Řezáč J., Hobza P. (2013). Competition between halogen, dihalogen and hydrogen bonds in bromo- and iodomethanol dimers. J. Mol. Model..

[46] Koopmans T. (1934). Über die Zuordnung von Wellenfunktionen und Eigenwerten zu den Einzelnen Elektronen Eines Atoms. Physica.

[47] Marchiori C.F.N., Carvalho R.P., Ebadi M., Brandell D., Araujo C.M. (2020). Understanding the electrochemical stability window of polymer electrolytes in solid-state batteries from atomic-scale modeling: the role of Li-ion salts. Chem. Mater..

[48] Adane L., Bharatam P.V. (2011). Computer-aided molecular design of 1H-imidazole-2,4-diamine derivatives as potential inhibitors of Plasmodium falciparum DHFR enzyme. J. Mol. Model..

[49] Nguyen V.-T., Huynh T.-K.-C., Ho G.-T.-T., Nguyen T.-H.-A., Le Anh Nguyen T., Dao D.Q., Mai T.V.T., Huynh L.K., Hoang T.-K.-D. (2022). Metal complexes of benzimidazole-derived as potential anti-cancer agents: synthesis, characterization, combined experimental and computational studies. R. Soc. Open Sci..

[50] Egbe D.A.M., Sell S., Ulbricht C., Birckner E., Grummt U. (2004). Mixed alkyl‐ and alkoxy‐substituted poly[(phenylene ethynylene)‐ *alt* ‐(phenylene vinylene)] hybrid polymers: synthesis and photophysical properties. Macromol. Chem. Phys..

[51] Subhash, Chaudhary A., Jyoti (2023). Synthesis, structural elucidation, DFT investigations, biological evaluation and molecular docking studies of tetraamide-based macrocyclic cobalt (II) complexes. J. Iran. Chem. Soc..

